# Chronic social isolation affects feeding behavior of juvenile zebrafish (*Danio rerio*)

**DOI:** 10.1371/journal.pone.0307967

**Published:** 2024-07-26

**Authors:** Aubrey Dissinger, Simona Rimoldi, Genciana Terova, Karolina Kwasek

**Affiliations:** 1 Department of Zoology, Southern Illinois University – Carbondale, Carbondale, Illinois, United States of America; 2 Department of Biological Sciences, University of New Hampshire, Durham, New Hampshire, United States of America; 3 Department of Biotechnology and Life Sciences, University of Insubria, Varese, Italy; Kafrelsheikh University, EGYPT

## Abstract

Many organisms exhibit social behaviors and are part of some scheme of social structure. Zebrafish are highly social, shoaling fish and therefore, social isolation may have notable impacts on their physiology and behavior. The objective of this study was to evaluate the effects of social isolation on feed intake, monoaminergic system related gene expression, and intestinal health of juvenile zebrafish fed a high-inclusion soybean meal based diet. At 20 days post-fertilization zebrafish were randomly assigned to chronic isolation (1 fish per 1.5 L tank) or social housing (6 fish per 9 L tank) with 18 tanks per treatment group (n = 18). Dividers were placed between all tanks to prevent visual cues between fish. Zebrafish were fed a commercial fishmeal based diet until 35 days post-fertilization and then fed the experimental high-inclusion soybean meal based diet until 50 days post-fertilization. At the end of the experiment (51 days post-fertilization), the mean total length, weight, and weight gain were not significantly different between treatment groups. Feed intake and feed conversion ratio were significantly higher in chronic isolation fish than in social housing fish. Expression of monoaminergic and appetite-related genes were not significantly different between groups. The chronic isolation group showed higher expression of the inflammatory gene *il-1b*, however, average intestinal villi width was significantly smaller and average length-to-width ratio was significantly higher in chronic isolation fish, suggesting morphological signs of inflammation were not present at the time of sampling. These results indicate that chronic isolation positively affects feed intake of juvenile zebrafish and suggest that isolation may be useful in promoting feed intake of less-palatable diets such as those based on soybean meal.

## Introduction

Many organisms exhibit social behaviors and there is a strong connection between social context and neural and cognitive function [1]. Social isolation has been shown to activate the hypothalamic pituitary adrenocortical/interrenal (HPA/I) axis and increase stress levels and feeding rate in a variety of organisms [2, 3]. Social isolation is often thought to induce a stress response in social animals, especially herding and shoaling species. However, social isolation may not be as stressful to some fish species as previously predicted. Multiple studies found either no change or reduced levels of cortisol in fish that were isolated compared to those in social housing (SH) [4–7]. Similar studies also showed a reduction in anxiety-related behaviors in isolated individuals [8, 9].

The monoaminergic system is involved in the regulation of physiological processes and has become increasingly important in understanding feeding and other behaviors in fish [10–12]. Two major neurotransmitters in the monoaminergic system are dopamine and serotonin. Dopamine is an important neurotransmitter when it comes to motivation and reward. It is largely involved in re-approach to previously rewarding stimuli and maintaining a pattern of behavior once reward is learned [13]. Serotonin is an important enteric signaling molecule involved in secretory functions in the gut [14] and plays a critical role in appetite and signaling satiety [15, 16]. Social interactions have been shown to increase monoamine levels: shoaling and presentation of conspecific images have been found to increase dopamine levels in zebrafish (*Danio rerio*) [17, 18] and social subordination can contribute to serotonin over-production [19]. Additionally, increases in dopaminergic and serotonergic activity have been associated with reduced feed intake [20–24]. Changes in production and release of these signaling molecules may help to understand the effects of social isolation on feed intake and feed utilization.

While most of the research on social isolation in fish evaluates stress response, neurochemical regulation, and immune response, little is known about how social isolation affects feeding and nutrition [5, 8–9]. This is critical considering that stress and hormone regulation play a large role in hunger cues and feeding rate in a variety of organisms [15, 25]. Social isolation has been shown to reduce appetite and feed intake in catfish [26, 27] and larval zebrafish [28]. However, there is also some connection between social isolation and increased feed intake. Multiple studies of mice and rats have found that those in isolation consume and weigh significantly more than those that are in social housing [29–31]. A study of isolated juvenile zebrafish found no significant difference in growth performance between isolated and social groups, suggesting no difference in feed intake throughout the study [5]. These contrasting results suggest that isolation could be a useful tool for gaining an understanding of how social context relates to feeding behavior.

Soybean meal (SBM) has become a common protein replacement for fishmeal (FM) in fish feed formulations because of its high protein content, high digestibility, and relatively well-balanced amino acid profile [32]. However, at high inclusion rates, SBM has been shown to negatively affect feed ingestion and growth rate and cause intestinal inflammation in a variety of species [32–37]. Understanding how to increase ingestion rates and utilization of dietary SBM without impacting health constitutes an important aspect of fish nutrition. Zebrafish have been found to have a middle-ground tolerance to SBM with no inflammatory effect detected up to 50% inclusion therefore, they could serve as models for testing methods of improving dietary SBM utilization [36].

Because social interactions have been found to increase monoaminergic activity and monoamines can have an inhibitory effect on feed intake, we hypothesized that fish in chronic isolation (CI) would have altered expression of dopamine and serotonin related genes, representative of reduced monoaminergic activity and thus resulting in increased feed intake when compared to SH fish. However, due to the inflammatory nature of SBM, increased feed intake of the experimental diet may lead to increased intestinal inflammation. Therefore, the objective of this study was to evaluate the effects of chronic social isolation on 1) feed intake and utilization of a high-inclusion SBM diet, 2) dopamine and serotonin-related gene expression, 3) appetite-related gene expression and 4) intestinal health using zebrafish as a model species.

## Methods

### Animal husbandry

The experiment was conducted in the Center for Fisheries, Aquaculture, and Aquatic Sciences at Southern Illinois University-Carbondale (SIUC), IL. All procedures were carried out in strict accordance with the recommendations in the Guide for the Care and Use of Laboratory Animals of SIUC. The SIUC Institutional Animal Care and Use Committee approved all protocols utilized during experimentation (Protocol # 21–006). Involved researchers were trained in accordance with SIUC Institutional Animal Care and Use Committee requirements. All efforts were made to minimize pain, stress, and discomfort in the experimental animals. Zebrafish were observed daily for signs of distress and discomfort and no fish had to be euthanized prior to experimental termination.

Wild-type juvenile zebrafish were housed in 1.5 L and 9.0 L polycarbonate aquaria on a recirculated aquaculture system (Pentair Aquatic Ecosystems, Cary, NC, USA) that was modified to be an open system with continuous flow-through of city water (Carbondale, IL, USA). The system was altered to prevent olfactory cues or hormonal signaling between tanks through shared system water [9]. City water was filtered through a carbon filter, aerated, and heated to 27.21 ± 0.24°C. Water pH and conductivity were monitored daily through the system and averaged 7.38 ± 0.09 and 189.23 ± 6.05 μS respectively. Dissolved oxygen was monitored daily using a multiparameter meter (YSI, Yellow Springs, OH) and averaged 6.72 ± 0.22 mg/L. Zebrafish were subject to a 10:14 photoperiod with automatic lights turning on at 08:00 hours and off at 18:00 hours. The illumination provided was at 245 lux and the distance between the surface of the water and the light source was 10 cm.

### Feed preparation

The experimental high inclusion SBM diet was formulated based on previous studies with some modification to allow for higher rates of SBM inclusion (55.7%) [36, 38, 39]. The diet was prepared at SIUC (Table 1). The dry ingredients were uniformly mixed using a mixer (Farberware, Fairfield, CA) adding water and oil components slowly until they were homogenous. The ingredients were then extruded (Caleva Extruder 20, Sturminster Newton Dorset, England), spheronized (Caleva Multibowl Spheronizer, Sturminster Newton Dorset, England), and freeze-dried (Labconco FreeZone 6, Kansas City, MO) to remove moisture. The diet was sieved to varying particle sizes; the particle size of feed was adjusted according to fish gape size throughout the experimental trial (150–355 μm).

**Table 1:** The feed formulation and proximate composition of the experimental diet (SBM: Soybean-meal-based diet).

Ingredients	SBM (%)
SBM^1^	55.70
Krill Meal^2^	10.00
CPSP^3^	5.00
Dextrin^4^	6.34
Fish Oil^5^	6.55
Soy Lecithin^6^	5.00
Mineral mix^7^	2.50
CaHPO_4_^4^	1.50
Vitamin mix^8^	2.50
Vitamin C^9^	0.10
Choline chloride^4^	0.06
Methionine^4^	0.50
Lysine^4^	1.00
Threonine^4^	0.25
Taurine^4^	1.00
CMC^10^	2.00
**Analyzed Composition (%)**	
Crude Protein	36.63 ± 0.12
Crude Lipids	15.43 ± 0.15
Ash	9.18 ± 0.02
Moisture	3.70 ± 0.02

^1^ Harper Feed Mill, Herrin, Il, USA

^2^ Processed *Euphausia superba*, Florida Aqua Farms, Dade City, FL, USA

^3^ Soluble fish protein hydrolysate, Sopropeche S.A., Boulogne Sur Mer, France

^4^ Dyets, Bethlehem, PA, USA

^5^ Cod liver oil, MP Biomedicals, Solon, OH, USA

^6^ Refined soy lecithin, MP Biomedicals, Solon, OH, USA

^7^ Bernhart-Tomarelli mineral mix with 5 ppm selenium in a form of sodium selenite, Dyets, Bethlehem, PA, USA

^8^ Custom Vitamin Mixture (mg/kg diet) Thiamin HCl, 4.56; Riboflavin, 4.80; Pyridoxine HCl, 6.86; Niacin, 10.90; D-Calcium Pantothenate, 50.56; Folic Acid, 1.26; D-Biotin, 0.16; Vitamin B12 (0.1%), 20.00; Vitamin A Palmitate (500,000 IU/g), 9.66; Vitamin D3 (400,000 IU/g), 8.26; Vitamin E Acetate (500 IU/g), 132.00; Menadione Sodium Bisulfite, 2.36; Inositol, 500, Dyets, Bethlehem, PA, USA

^9^ L-Ascorbyl-2-Polyphosphate, Argent Aquaculture, Redmond, WA, US

^10^ Carboxymethyl cellulose sodium salt high viscosity, MP Biomedicals, Solon, OH, USA

### Brood-stock spawning and larval fish

Wild-type juveniles were spawned from a brood-stock composed of domesticated individuals that originated from pet store zebrafish stocked to the SIUC system in 2018 (Petco, Carbondale, IL, USA). Males and females were separated for a week and fed twice a day with *Artemia* nauplii and commercial diet (Otohime, Japan). After this separation period, males and females were combined in a 9L tank to breed and a mesh insert on the tank bottom was used to protect fertilized eggs from adults. Larval fish were reared in a “common garden” in the 9L tank on the modified flow-through system until 20 days post-fertilization (dpf). As zebrafish do not exhibit shoaling behavior until 7 dpf, extended time together allowed zebrafish to become acclimated to social groups and exhibit social behavior [9]. Zebrafish were fed an abundance of rotifers (*Brachionus plicatilis*) as a first feeding for 4 days followed by *Artemia* nauplii for 10 days [40]. Once able to be weaned to dry feed, larval fish were trained on a commercial diet (Otohime, Japan).

### Experimental design and feeding regimen

**Fig 1:**
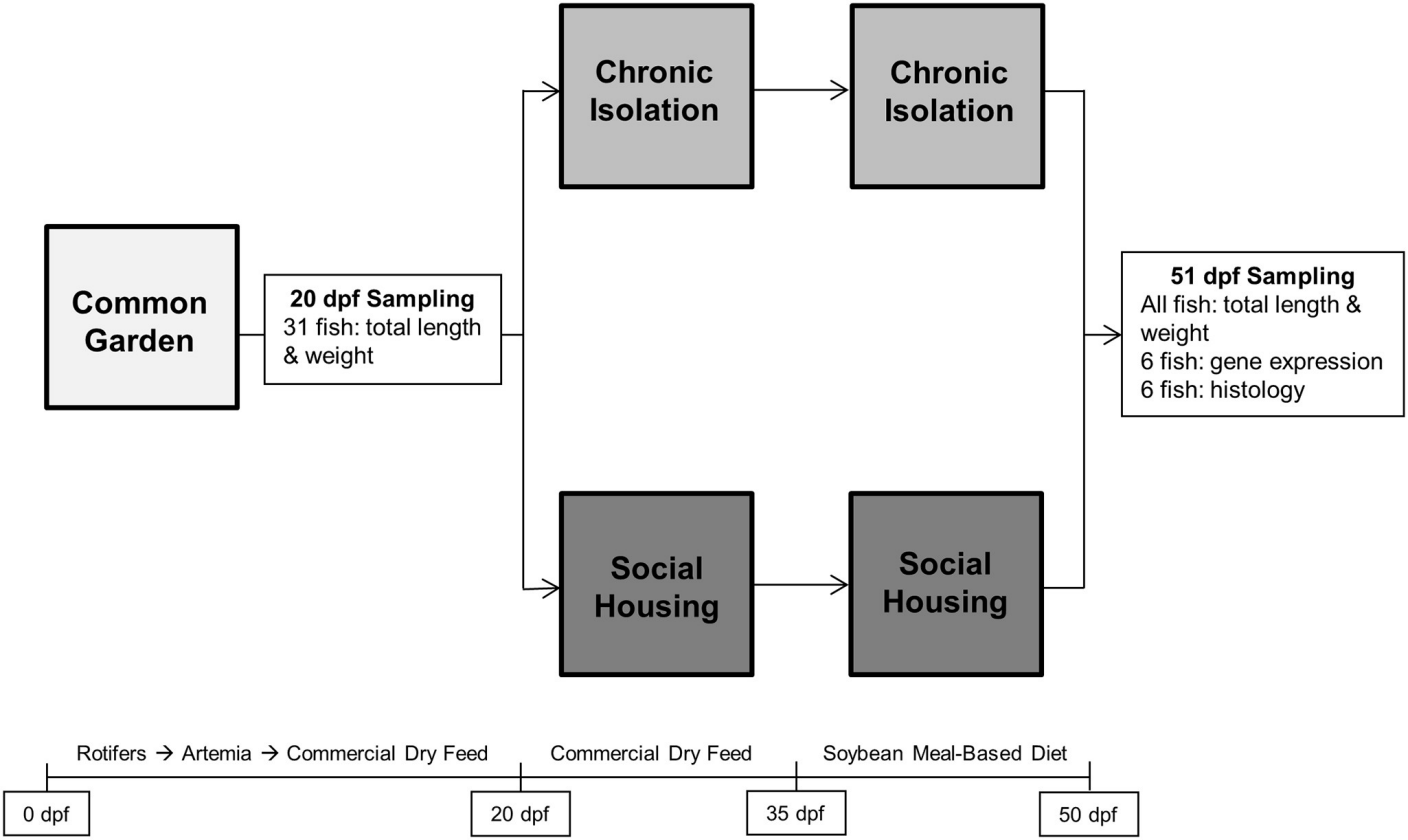
Experimental design displaying the timeline for separation into treatment groups, feeding regime, and sampling schedule (dpf = days post-fertilization).

To avoid any biases derived from sexual dimorphism of zebrafish, the experiment was carried out from 20 to 51 dpf, before zebrafish reached sexual maturity. At 20 dpf, zebrafish were separated into one of two treatment groups: SH or CI with 18 tanks per treatment group (n = 18, Fig 1). Social housing was used as the control group as this method of housing is most commonly used in zebrafish husbandry. Density was controlled for in each experimental group by stocking one fish per 1.5 L of water. Consequently, zebrafish assigned to CI were housed in 1.5 L tanks with one fish per tank and zebrafish assigned to SH were housed in 9 L tanks with six fish per tank. Additionally, water flow was set to 70 ml/min in the 1.5 L tanks and 420 ml/min in the 9 L tanks to maintain the same exchange rate of water between treatment groups. To randomize treatment assignment, fish from the common garden were stocked into treatment tanks one at a time, rotating between SH and CI tanks. Once the CI tanks were filled, fish stocking continued one fish at a time in the SH tanks until they were filled. As visual cues from conspecifics are important for developing learned social behavior, black tarp dividers were installed between all tanks to prevent visibility between them [9].

Once separated into treatment groups (CI or SH), fish were fed commercial diet (Otohime, Japan) three times per day to apparent satiation for 15 days ensuring that CI fish were exposed to chronic isolation before introduction of the experimental diet. After this 15-day acclimation period [4] in treatment tanks (35 dpf), the experimental SBM-based diet was introduced. All zebrafish were fed the high-inclusion SBM diet to apparent satiation three times per day for another 15 days. Each feeding period was limited to 1 h of satiation feeding–the three feeding periods were 08:00–09:00, 12:00–13:00, and 16:00–17:00. Zebrafish were initially offered a small amount of food and their feeding behavior was carefully observed. Tanks were provided additional small amounts of feed if “hunting” behavior was observed, and fish were monitored to ensure these offerings were consumed. Once signs of slowed feeding behavior were observed, feed offerings were halted. To accurately compare feed intake between treatment groups, feeding ceased when the single fish in CI tanks discontinued feeding behavior and when only one fish remained feeding in SH tanks, or when the 1 h feeding period ended. Diets for each tank were weighed before and after daily feedings to measure the amount of feed consumed daily.

### Sampling

Due to their small size and to avoid injuries or mortalities of fish utilized in this study, three randomly collected subsamples of 10–11 zebrafish from the common garden (n = 31) were measured to obtain weights and lengths prior to stocking the treatment tanks at 20 dpf. The sample fish were euthanized in an ice bath, patted dry, and individually weighed and measured for total length. The average weight and total length of the 31 samples was used as an average initial weight (8.63±2.57 mg) and length (9.93±0.81 mm) of juvenile fish stocked into treatment tanks.

At the end of the experiment (51 dpf), all zebrafish were moved to petri dishes and photographed using a Sony Cybershot DSC RX100 III digital camera. ImageJ (NIH Image) was used to determine the length of all individual fish in each treatment group. Fish were patted dry and weighed using an analytical scale (Mettler-Toledo, Columbus OH, USA). Twelve fish from the 18 tanks in each treatment group were randomly selected and euthanized using ice slurry with equal proportions of water and ice [41]. To accurately compare the treatment groups, one fish from SH was compared to one fish from CI. From the samples in each treatment group, six were randomly assigned for analysis of gene expression and the remaining six for histological analysis. For the zebrafish assigned to gene expression, the digestive tract and head were dissected and stored in RNAlater (Sigma-Aldrich, St. Louis, MO, USA). For the zebrafish assigned to histology, bodies were bisected at an angle below the anal fin and stored in formalin. Growth performance was measured as total length and weight. Weight gain was calculated by subtracting the average initial weights obtained from the common garden (W_0_) from the final weights (W_f_). Feed intake was measured daily and feed conversion ratio (FCR) was calculated by dividing cumulative feed intake by weight gain.

### Gene expression analysis

Dissected zebrafish heads and bodies were stored in RNAlater at -20°C after sampling. Samples were manually homogenized and processed using TRIzol Reagent (Ambion, Foster City, CA, USA) and RNA was extracted using the On-Column PureLink^™^ DNase Treatment (PureLink^™^ RNA Mini Kit and PureLink^™^ DNase, Invitrogen, Carlsbad, CA, USA) following the manufacturer’s instructions. Once the RNA was extracted and purified, each RNA sample was quantified (ng/μl) using a spectrophotometer (Nanodrop 2000c, Thermo Fisher Scientific, Waltham, MA, USA). Then, 500 ng of RNA from each sample was reverse transcribed using the High-Capacity cDNA Reverse Transcription Kit (Applied Biosystems, Foster City, CA, USA). The RT-reactions were then diluted (1:10) and 4 μl of each diluted cDNA were used in a 25 μl volume of quantitative PCR (qPCR) reaction. Gene expression analysis was performed using a CFX96 Connect^™^ Real-Time PCR Detection System (Bio-Rad) and iTaq Universal SYBR^®^ Green Supermix (Bio-Rad). Primers (Table 2) were designed using the open source Primer3 program under the following settings for best qPCR efficiency: mplicon size 70–150 bp; primer melting temperature 60°C; GC primer content > 50%. All assays showed an efficiency between 87–105%.

**Table 2:** Primers used for gene expression analysis.

Gene	Gene Description	Primers for Semi-Quantitative RT-PCR	GenBank Accession Number
*drd1*	Dopamine receptor D1	**F:** GGAGGACGACTCTGGCATAA **R:** AGACGTCCGCATCACTATCC	FJ208849.1
*drd2a*	Dopamine receptor D2a	**F**: GCAACACCAGCAGTATCACG **R**: GGCCTTCTTCTCCTTCTGCT	NM_183068.1
*drd3*	Dopamine receptor D3	**F**: CCTGCCCTCTACTGTTTGGA **R**: AACGACACCACGGAGGAGTA	NM_183067.1
*drd4a*	Dopamine receptor D4a	**F**: GCAAGCTGGAGGACAACAAC **R**: GCAGTACAGGAGCAGCATGA	NM_001012616.3
*htr1bd*	Serotonin receptor 1bd	**F**: CTCACACGCAAGCTTCACAC **R**: TGGACACCAGGAGGTCTGTC	NM_001145686.1
*mao*	Monoamine oxidase–metabolism of biogenic amines	**F**: CGAGTCAGATCTGGCAGTCA **R**: TTCTCTCCCAGAAGGTGGTG	NM_212827.3
*tph1a*	Tryptophan hydroxylase 1a –synthesis of serotonin	**F**: GGAGCATCAGACGACTCCAT **R**: TTACAGAGCCCGAACTCCAC	NM_178306.3
*slc6a4a*	Sodium-dependent serotonin transporter	**F**: TGCCAGCTACAATCCCTTTC **R**: AAGCCTGACAGGAAACTGGTC	XM_009291668.3
*slc18a2*	Vesicular monoamine transporter 2	**F**: AGTGCTTGATGGAGCTCTGC **R**: GGAACCTGCTGCAATGAGG	NM_001256225.2
*il-1b*	Interleukin-1 beta–pro-inflammatory cytokine	**F**: TGGACTTCGCAGCACAAAATG **R**: GTTCACTTCACGCTCTTGGATG	NM_212844.2
*il-6*	Interleukin 6 –pro-inflammatory cytokine	**F**: GCGTCCTGACGTGGTATAAAG **R**: GTCGTTTGGTGCTGTGTTTG	JN698962.1
*tnfa*	Tumor necrosis factor alpha–pro-inflammatory cytokine	**F**: GCGCTTTTCTGAATCCTACG **R**: TGCCCAGTCTGTCTCCTTCT	NM_212859.2
*agrp*	Agouti related neuropeptide–orexigenic effects	**F**: GTCCACCTGCAGAGAAGAGG **R**: GCCTTAAAGAAGCGGCAGTA	NM_001328012.1
*galr1b*	Galanin/GMAP receptor–orexigenic effect	**F**: CACATGCTGTTATGCCAAGGT **R**: CAACACCGTCTGAGCTGTCT	NM_001327843.1
*ghsra*	Ghrelin receptor–orexigenic effect	**F**: TGCCTGTGTTCTGCTTAACTGTC **R**: ACACCACCACAGCCAGCAT	NM_001146272.1
*nucb2b*	Nucleobindin 2 –anorexigenic effect	**F**: GGGCTTGTTTGGATGCACTG **R**: GCCGGTGTCTGCATTTTCAG	NM_201493.1
*npy*	Neuropeptide Y–orexigenic effect	**F**: TGGGGACTCTCACAGAAGGG **R**: AATACTTGGCGAGCTCCTCC	BC162071.1
*ghrl*	Ghrelin–orexigenic effect	**F**: GTGTCTCGAGTCTGTGAGCG **R**: CAGCTTCTCTTCTGCCCACT	AM055940.1
*lep*	Leptin a–anorexigenic effect	**F**: TGTTGACCAGATACGCCGAG **R**: GTCCAGCGCTTTCCCATTTG	NM_001128576.1
*cck8*	Cholecystokinin–regulating the release of digestive enzymes	**F**: GTTCAGTCTAATGTCGGCTCC **R**: TAGTTCGGTTAGGCTGCTGC	NM_001386383.1
*eef1a1*	Eukaryotic translation elongation factor 1 alpha 1	**F**: GCTGGCAAGGTCACAAAGTC **R**: GAAGAACACGCCGCAACCT	NM_131263.1

The qPCR protocol consisted of an initial denaturation step of 95°C for 30 s, followed by 40 cycles of denaturation for 5 s at 95°C and annealing/extension for 30 s at 60°C. The efficiency of PCR reactions was higher than 90% and negative controls without sample templates were performed for each primer set. The specificity of PCR reactions was verified by analysis of melting curves (ramping rates of 0.5°C/10 s over a temperature range of 65–95°C). Fluorescence raw data were collected during the PCR extension phase and analyzed by Bio-Rad CFX Maestro software. The ΔΔCt method [42] was applied to calculate the relative gene expression levels, using eukaryotic translation elongation factor 1 alpha 1 (eef1a1) as housekeeping gene. Fold-change variation for each gene was expressed referred to SH control group (values >1 indicate up-regulated genes, conversely values < 1 indicate down-regulated genes).

### Histological analysis

Slides for histological analysis were prepared at Saffron Scientific Histology Services (Carbondale, IL). Zebrafish samples were cut below the anal fin to open the body cavity and fixate the intestine in 10% neutral buffered formalin. Samples were moved through increasing concentrations of ethanol until all water was removed from the tissue. Ethanol was then replaced with paraffin wax, infiltrating the tissues. Samples were embedded in a paraffin wax block, sectioned at 5μm intervals, and stained with hematoxylin and eosin (H&E). Slides were viewed at 100x magnification using a microscope (Nikon SMZ1500) and pictures were taken using Nikon Digital Sight. Only three samples from each group were able to be analyzed due to the destruction of intestinal villi through the mounting process. Six intestinal villi from each slide were randomly selected for measurements. Intestinal villus length and width were measured using NIS Elements software (Nikon Instruments Inc., Melville, NY).

### Statistical analysis

Statistical analyses were performed using R software [43]. T-tests were used to compare average length, weight, feed intake, FCR, gene expression levels, and intestinal villi (length, width, and length:width ratio) between treatment groups. All data were normal; Welch’s t-tests rather than Student t-tests were used in the case of unequal variances in data. Differences between groups were considered significant at p values < 0.05.

Mixed model repeated measures analysis was used to compare FM intake between treatment groups for the first 15 days of the feeding trial. A logarithmic transformation was used on the feed intake data to meet the homogeneity of variances assumption for this analysis. Akaike’s Information Criterion (AIC) model selection was used to select the best fit correlation structure; first order autoregressive correlation was used for this data set. Differences between groups were considered significant at p values < 0.05.

## Results

### Growth performance

**Fig 2:**
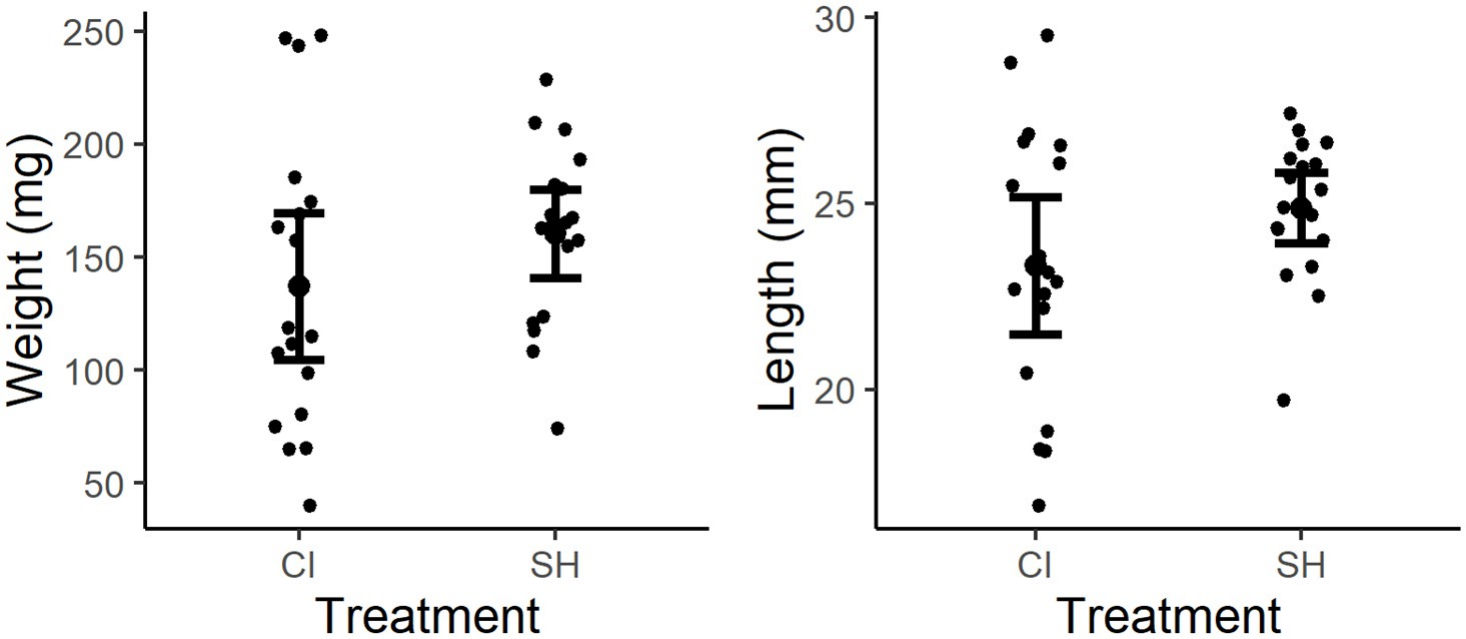
Growth performance metrics represented as final weight (CI: 137.1±65.1 mg, SH: 160.3±39.2 mg) and total length (CI: 23.3±3.73 mm, SH: 24.9±1.9 mm).

At the end of the experiment, the mean weight and weight gain were not significantly different between treatment groups (p > 0.05). Nor was the mean total length significantly different between treatment groups (p > 0.05; Fig 2).

### Feed utilization

**Fig 3:**
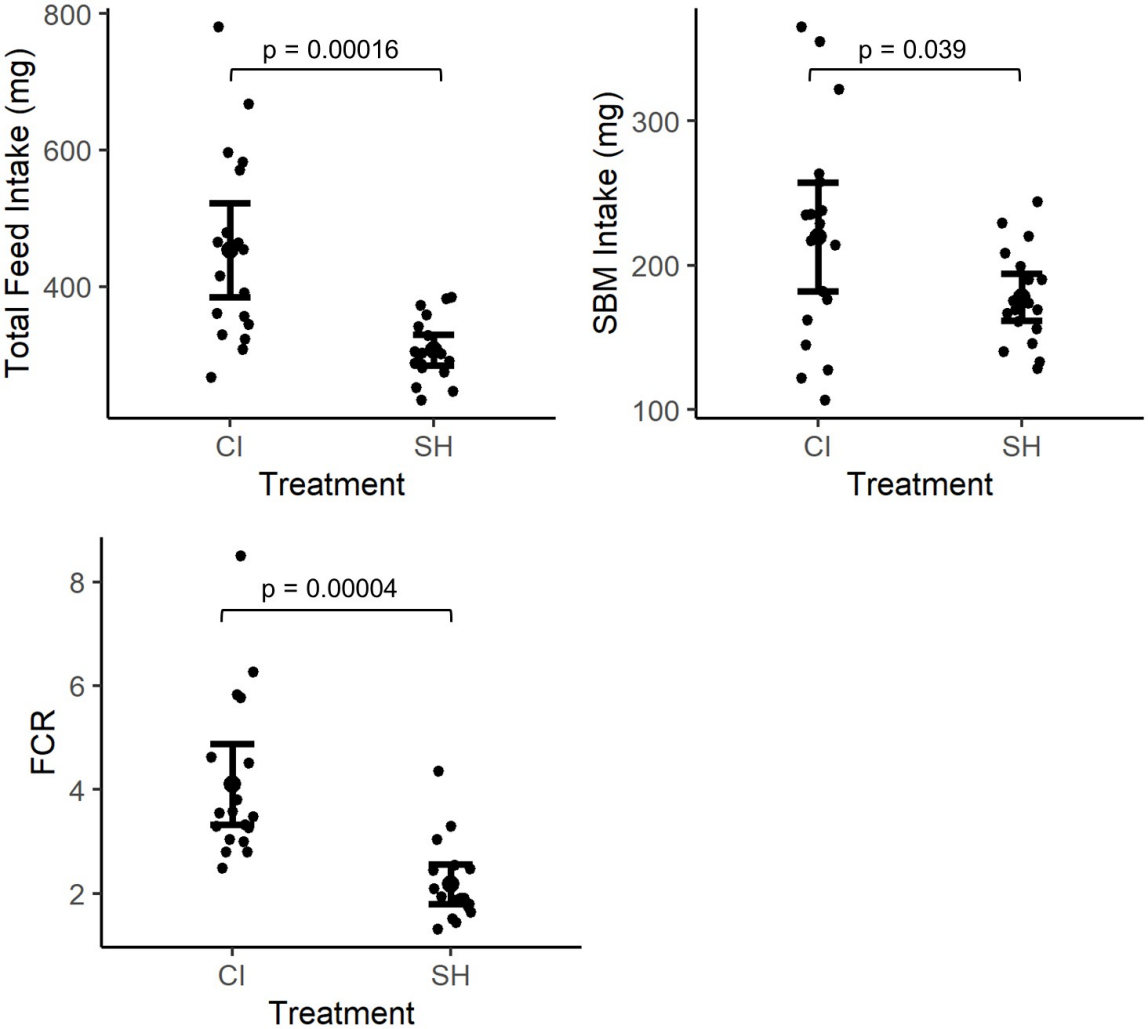
Feed utilization metrics represented as a) total feed intake (commercial + SBM; CI = 453.5±138.9 mg, SH = 307.0±45.6 mg), b) SBM intake (CI = 219.7±75.8 mg, SH = 177.9±32.9 mg), and c) feed conversion ratio (FCR; CI = 4.11±1.56, SH = 2.18±0.76). Feed intake was measured in milligrams per fish for both treatments throughout the study. Brackets with p-values indicate significant difference (p < 0.05).

Total feed intake (commercial plus SBM-based diet) measured as average milligrams of feed consumed per fish was significantly higher in CI fish than in SH fish (t = 4.25, df = 34, p = 0.00016) over the course of the study. Soybean meal-based diet intake over the 15-day experimental feeding trial was also significantly higher in CI fish than in SH fish (t = 2.1471, df = 34, p = 0.039). While feed intake of commercial diet was higher on average in CI fish compared to SH fish, no significant differences were detected in average total commercial diet intake between treatment groups (p > 0.05). The FCR was significantly higher in the CI group than in the SH group too (t = 4.7138, df = 34, p = 0.00004). Feed intake and FCR are presented in Fig 3.

**Fig 4:**
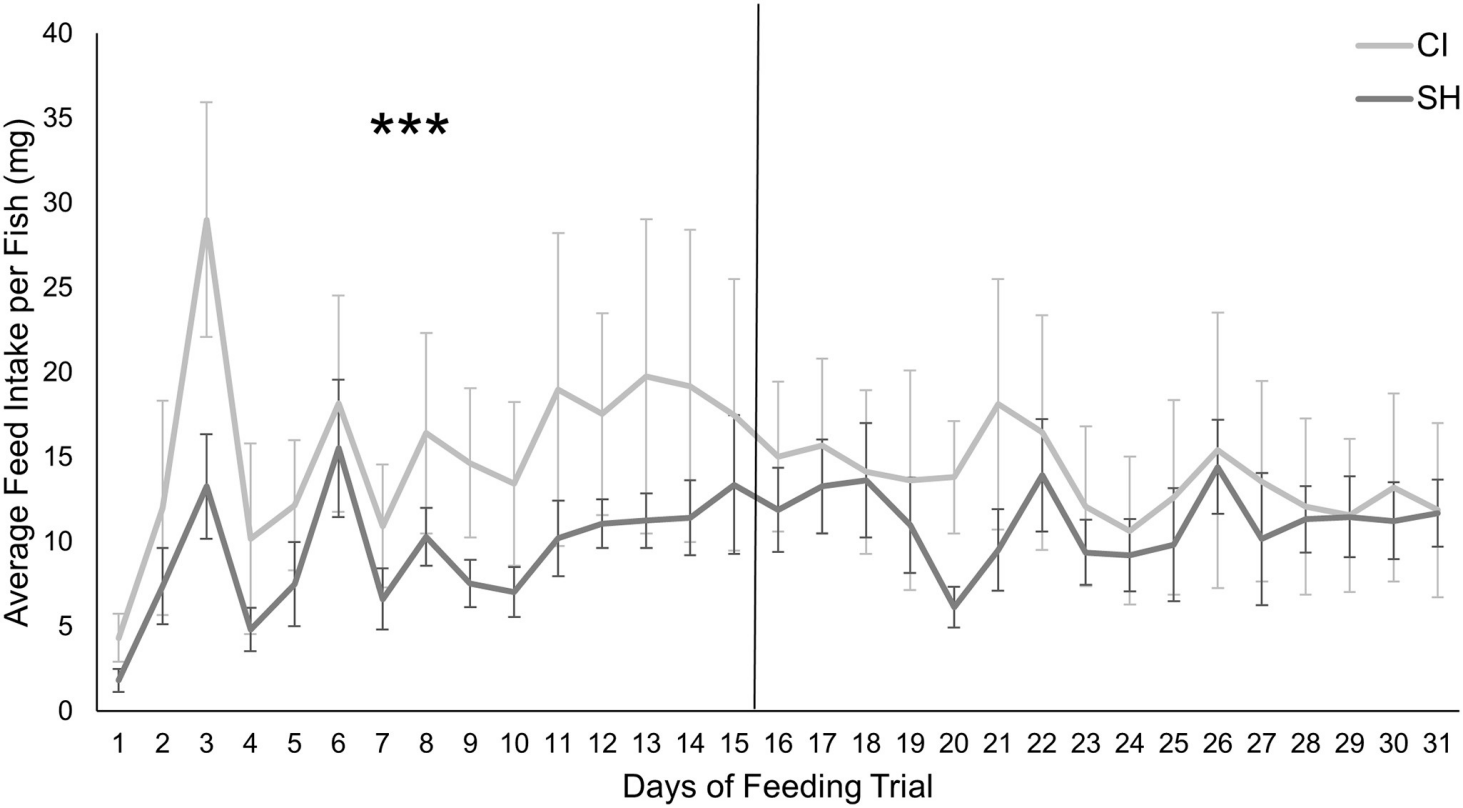
Average daily feed intake of FM-based diet and SBM-based diet per fish (mg) over time. The line indicates separation of FM and SBM feeding periods. The asterisks (***) indicate a highly significant effect of both treatment and time during the FM feeding period.

With an increase in age, there was some increase in average feed intake of the commercial diet, but once SBM was introduced, there was a gradual decline in daily feed intake for both treatment groups (Fig 4). Repeated measures analysis indicated a highly significant effect of both main effects (treatment and time) as well as the treatment by time interaction (p < 0.0001). When examined as a percentage of biomass, commercial feed intake on the first day of the experiment (20 dpf) was significantly higher in the CI group (50.08 ± 16.39) than the SH group (20.96 ± 7.72; t = 6.8158, df = 34, p < 0.0001). However, on the final day (50 dpf), SBM diet intake as a percent of biomass was not statistically significant (p > 0.05) between the two groups (CI = 9.38 ± 3.18, SH = 8.57 ± 1.27).

### Gene expression

**Fig 5:**
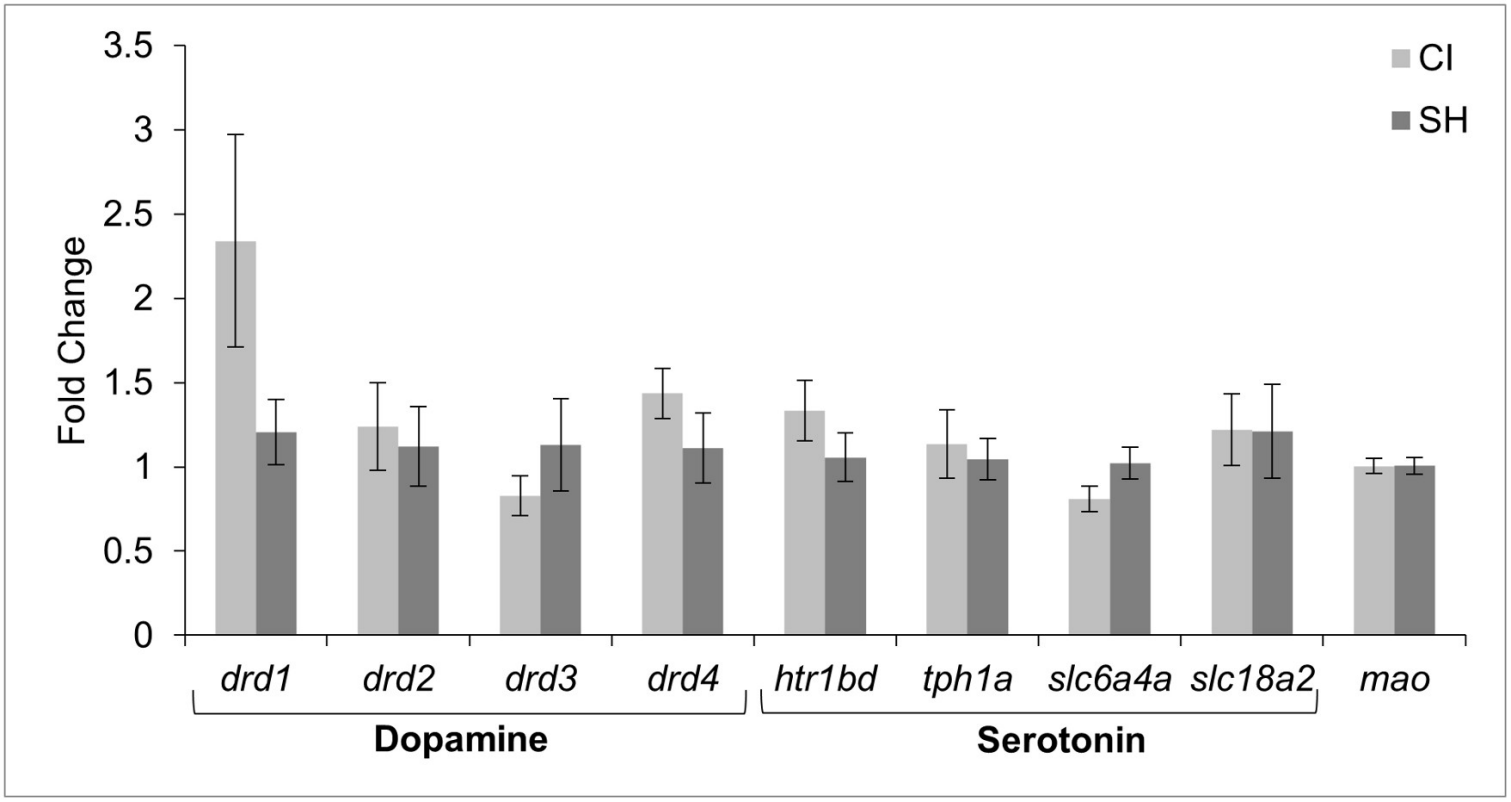
Expression of neurotransmitter-related genes in the zebrafish brain represented as fold change. Values are presented as mean fold change ± standard error of mean.

Genes involved in dopamine and serotonin transport, reception, and metabolism were analyzed (Fig 5). There was no significant difference in expression of any of the monoaminergic-related genes between treatment groups.

**Fig 6:**
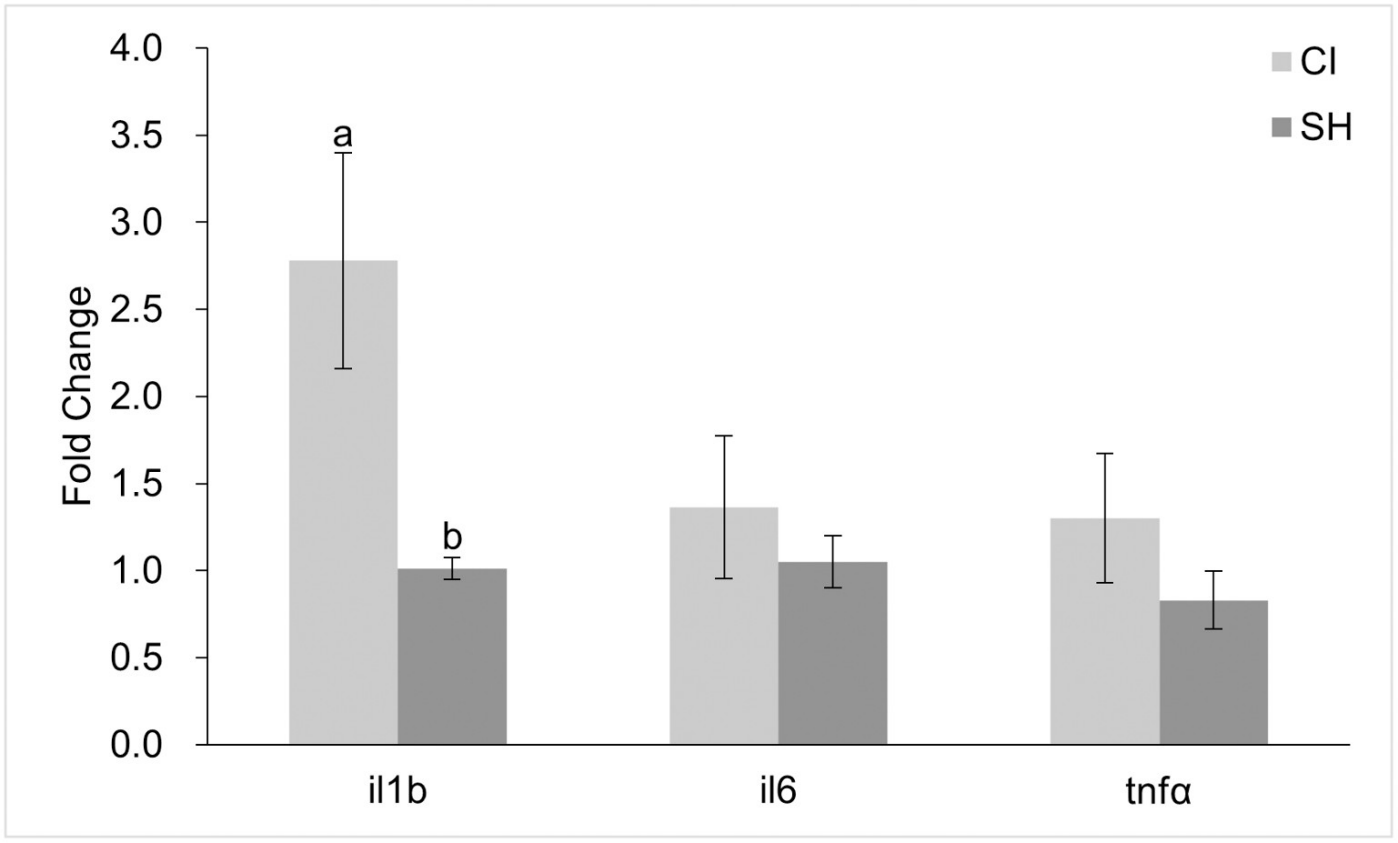
Expression of inflammation-related genes in the zebrafish intestine represented as fold change. Values are presented as mean fold change ± standard error of mean. Significant differences between groups (p < 0.05) are indicated by different letters.

Inflammation-related genes in the gut were also analyzed (Fig 6). The CI group showed a significantly higher expression of *il-1b* than the SH group (p = 0.004327). There was no significant difference in the expression of *il-6* or *tnfa*.

**Fig 7:**
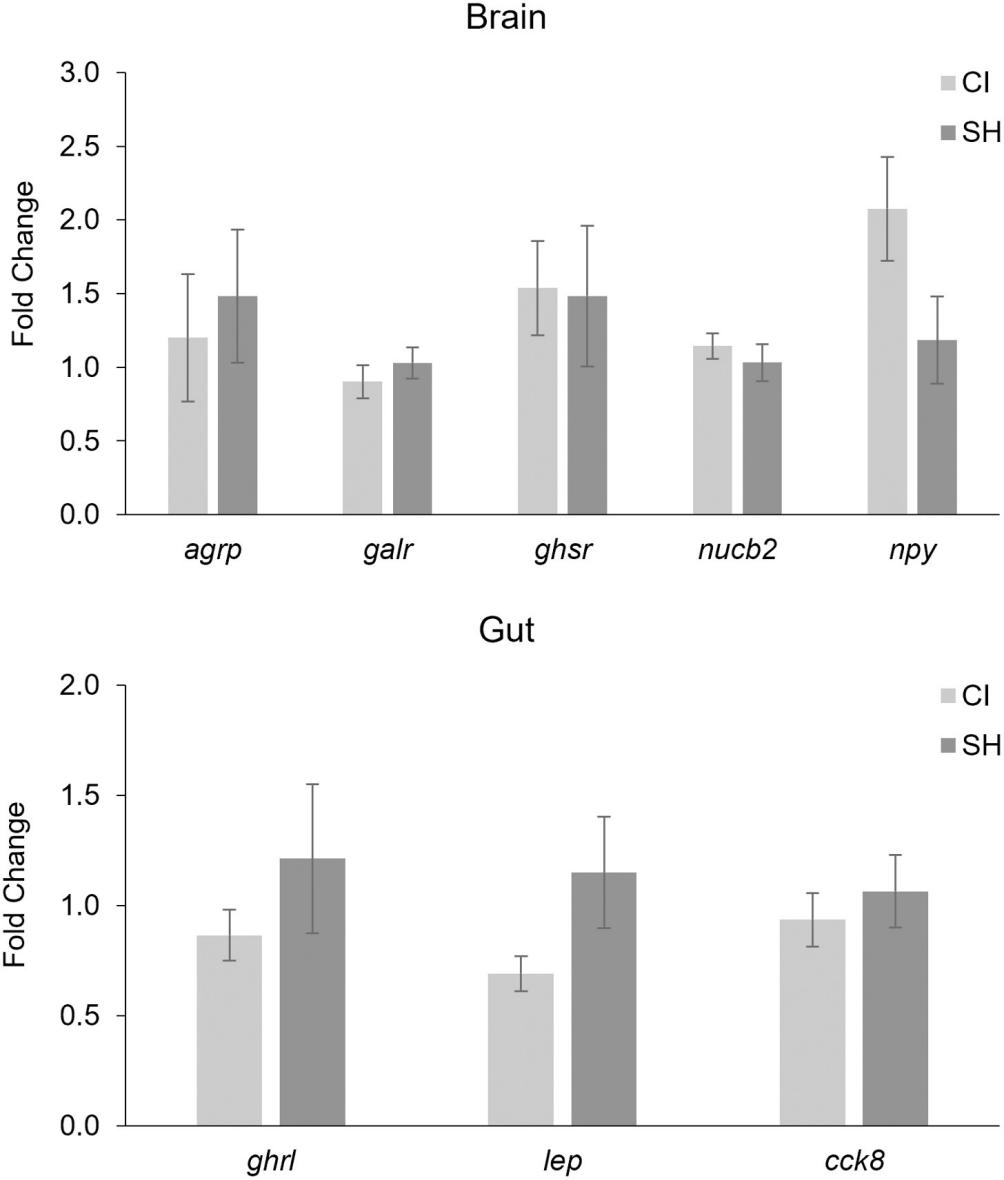
Expression of appetite-related genes in the zebrafish brain and intestine represented as fold change. Values are presented as mean fold change ± standard error of mean.

Analysis of appetite-related genes was performed in both the gut and the brain (Fig 7). There was no significant difference in any of the appetite-related genes; however, expression of ghrelin, leptin, and cholecystokinin was numerically higher in the SH group than in the CI group.

### Histological analysis

**Fig 8:**
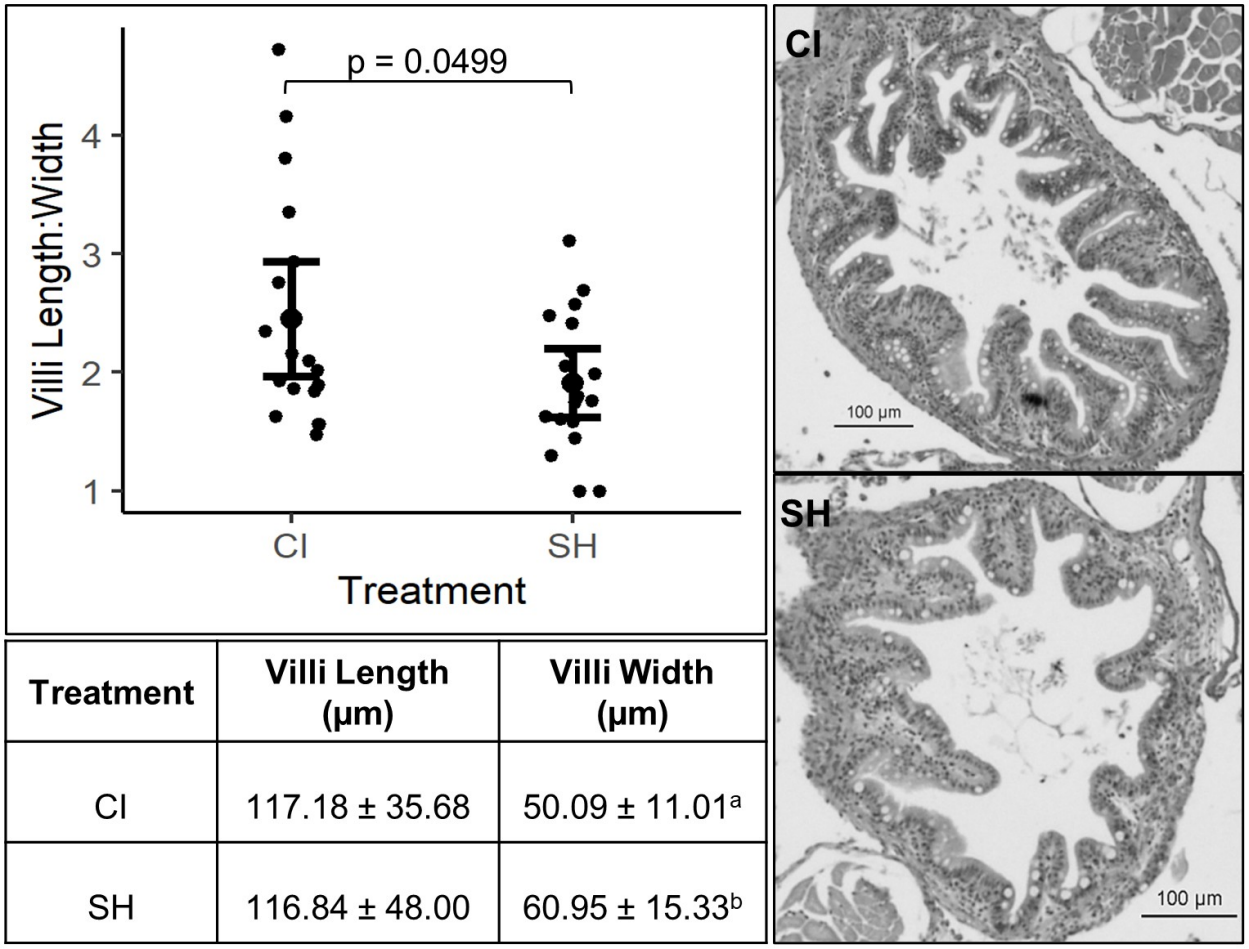
Measurements of distal intestinal villi length, width, and length-to-width ratio. Brackets and letters indicate significant difference between treatment groups (p < 0.05). Images show the distal intestine of chronically isolated (CI) and socially housed (SH) fish.

The distal portion of the intestine was used for histological analysis as diet-related inflammation is known to be present in this region [35, 36]. The intestinal villi length was not significantly different. However, the intestinal villi width was significantly different between treatment groups (t38 = -2.1082, p = 0.04166), the average width being higher in the SH group than in the CI group. Intestinal villi length-to-width ratio was measured by dividing each villi length by its width. The length to width ratio of intestinal villi was significantly higher in the CI group (2.45 ±0.97) than in the SH group (1.91 ±0.58; t34 = 2.0327, p = 0.0499; Fig 8).

## Discussion

During early development, social organisms learn by observing conspecifics and the social environment of an individual can largely shape brain development and behavior [5, 9, 44]. Social isolation is often thought to induce a stress response in social animals [2, 45], and can result in changes in both physiology and behavioral phenotype [46]. The results from this study showed no effect of social isolation on growth performance or monoaminergic system gene expression; however, social isolation appears to have a notable effect on feeding behavior.

Consistent with previous reports of zebrafish [5, 9], this study found no significant differences in weight or total length between CI and SH treatment groups. This suggests that isolation itself does not appear to have a negative impact on growth performance of the species. A similar result was found in a study of adult male medaka (*Oryzias latipes*) with no difference in final body weights between isolated or group housed fish after a 2-week period [47].

In contrast, studies of juvenile catfish (*Clarius gariepinus*) found significantly reduced feed intake and growth performance in individually housed fish compared to those that were socially housed [26, 27]. This was seen in a study of juvenile cichlids (*Pelvicachromis taeniatus*) as well: fish reared in isolation were significantly smaller than those in social groups as well [48]. Interestingly, the smallest fish in the social housing treatment group were significantly smaller than the isolated fish, suggesting that subordination can alter growth performance in social groups [48].

The variable effect of social isolation on growth may be linked to predation, social status, and competition [48]. Shoaling is an effective strategy against predation, providing valuable benefits for survival [49]; however, there are also costs to shoaling, including competition for resources and transmission of diseases [50]. Thus, in the absence of predation, as in typical fish culture and laboratory systems, the benefit of group living may not be as pronounced, resulting in variable growth performance [50]. As an example, juvenile nine-spined sticklebacks (*Pungitius pungitius*) descending from low-predator density pond populations grew significantly more when socially isolated than those that were group-housed [50]. Additionally, dominant individuals in a shoal are better able to monopolize a food source and tend to grow much faster than their subordinate counterparts, suggesting that social status and competition can result in a high variation in size within a group [48].

A common indicator of stress in fish is reduced appetite, leading to decreased feed intake, feeding efficiency, and growth [25]. However, the appetite regulation response may differ for acute versus chronic stressors [51–53]. While it is widely assumed that isolation would increase stress levels, length of isolation may greatly affect stress regulation and coping mechanisms in fish. In contrast to chronic isolation in which a fish may become adapted to their social context over time, acute isolation may increase stress levels as a sudden change in environment and social context may be alarming. Habituation and suppression of stress response may occur only after frequent exposures to a particular stressor [53]. It has been shown across multiple studies that CI or chronic stress in isolated zebrafish led to decreased levels of cortisol and stress-related behavior compared to those that were acutely isolated [54] or SH [4, 9, 54]. The zebrafish in this study were in isolation for 30 days and previous studies have considered acclimation to isolation or “CI” to be anywhere from 15 to 90 days [4, 8]. It is possible that fish exposed to CI in this study were less stressed after acclimating to their social environment and consequently consumed more feed. Though, the stress status of the CI fish is speculative and based on the observed feeding behavior as stress levels were not quantified in the present study. However, this potential ability for coping with isolation can also be seen in angelfish (*Pterophyllum scalare*). Feeding rates of angelfish that were kept in isolation and transferred to a new tank were significantly higher than those of individuals isolated for the first time. This finding suggests that the long-term isolated fish may adjust to the absence of conspecifics over time [55].

Considering that feed intake is a large determining factor in growth performance, and that CI fish consumed significantly more feed throughout this study, the similar growth performance between groups was perplexing. Increased feed intake but no increase in weight explains the significantly higher FCR in the CI group compared to that of the SH group. Japanese seabass (*Lateolabrax japonicas*) fed a 50% SBM diet showed a significantly higher feed intake than those fed a FM diet [56]. The 75% SBM group had the second highest feed intake of the three diets, although not significantly different from the FM group. However, feeding efficiency in both SBM groups was significantly reduced compared to the FM group [56]. Similarly, a study of juvenile tilapia (*Oreochromis niloticus* × *O*. *aureus*) saw an increasing trend in feed intake with increasing levels of dietary SBM inclusion [57]. The 100% SBM group presented with the highest feed intake and FCR [57]. The reduced utilization of SBM diets even at higher feeding rates is most likely attributed to the low digestibility of SBM-based diets and the presence of anti-nutritional factors [32, 36, 56, 58]. Therefore, the increased feed intake of diets high in SBM often reflects compensation for the reduced digestibility and nutrient uptake [59].

Stress creates communication between the brain and body; the hypothalamic pituitary adrenocortical or interrenal (HPA/I) axis tells the cardiovascular and immune systems how to respond via neural and hormonal mechanisms [51]. Social isolation has previously been shown to cause increased activation of the HPA/I axis in social animals [2, 6]. Increased activation in early life can lead to dysfunction of the HPA/I axis, which can then cause inflammation, impair the immune system [5, 60, 61], and induce a higher prevalence of depression and anxiety-related disorders [62, 63]. However, previous studies of zebrafish found varying physiological and behavioral responses to social isolation including no change or reduced cortisol [4–7] and anxiety levels [8, 9] suggesting that the relationship between social environment and stress is more complex in this particular species.

Shoal cohesion has been found to increase with age in zebrafish and similarly, dopamine and its metabolite 3,4-Dihydroxyphenylacetic acid (DOPAC) levels also exhibit age-dependent increases [17, 64]. Therefore, it may be expected that a socially isolated zebrafish that is unable to shoal would present with reduced dopaminergic activity. This effect was observed in studies of isolated zebrafish in which either reduced DOPAC [9] or a reduction in both DOPAC and dopamine were observed [54]. However, our gene expression results indicated that there was no significant difference in the expression of dopamine receptors D1-D4 between CI and SH fish. This finding suggests that there was no difference in dopamine activity between treatment groups. Similar results were found in other studies of isolated versus socially housed zebrafish [8, 65].

One possible explanation for the similar expression of dopamine-related genes between treatment groups could revolve around feed intake and hyperactivity. Motivation to eat and consumption of food activates dopamine production and release; repeated activation of the dopaminergic system through consumption of food strengthens the habit and reinforces the behavior [13, 66]. Chronically isolated fish consumed significantly more on average than those in the SH group. In addition, the CI fish quickly adjusted to a routine of feeding and appeared to respond to subtle cues of meal-time more readily. Similarly, exercise and locomotion are also linked to dopaminergic activity [67–71]. In a study of zebrafish with pharmacologically induced depression, dopamine levels were significantly reduced [72]. However, when these zebrafish were challenged with exercise for 20 days, there was a significant rise in dopamine, suggesting that physical exercise was able to combat depression-induced dopamine dysfunction [72]. Although not measured in the present study, CI fish appeared to have more frequent bouts of hyperactivity and swimming behavior than their SH counterparts. Perhaps the increased feed intake and hyperactivity observed in this study aided in offsetting the decrease in dopaminergic activity previously recorded in isolated zebrafish [9, 54].

Social stress and social subordination have been shown to cause chronic activation of the serotonergic system in adult zebrafish [73]. In addition, socially subordinate fish characteristically show a reduction in locomotor behavior and feed consumption when in the presence of conspecifics [73]. As social stress can contribute to serotonin over-production, we expected to see lower serotonergic activity in the socially isolated fish than in social groups. However, we found no significant difference in gene expression of serotonin receptors and transporters. Past studies of zebrafish have found varying impacts of social isolation on serotonin and its metabolite (5H1AA) with no change in either, reduction in serotonin only, or reduction in 5H1AA only [8, 9, 54]. One aspect to consider is that serotonin synthesis is dependent on tryptophan availability and thus diet and feed intake may be involved in serotonin production as well [73–76].

Overall, monoamine-related gene expression did not give a clear insight into the mechanisms behind the increased feed intake observed in the fish that were socially isolated. However, the monoaminergic system is complex and can be influenced by a wide range of factors [77–79], and gene expression analysis does not provide the full context in which this system functions. Thus, we cannot fully rule out that these neurotransmitters played a role in the results of this study.

Measuring cortisol is a widely approved method for determining stress levels in fish and characterizing the coping abilities of fish in response to novel environments [80]. As a non-invasive method of measuring this hormone [80], we collected water samples throughout the study in an attempt to evaluate how stress levels varied across social treatments. However, we were unable to extract quantifiable levels from these water samples. In the future, improving this method of collection and quantification could provide valuable insights into how fish adapt to social treatments over time.

Feed intake is regulated by complex gut-brain neuroendocrine interactions [81]. Appetite-related genes, namely those involved in orexigenic and anorexigenic signaling, were analyzed due to their involvement in regulating feed intake [82]. While there were no significant differences in the expression of any of the appetite-related genes analyzed, it is worth noting that SH fish displayed numerical upregulation of ghrelin, leptin, and *CCK* transcripts compared to the CI fish.

Ghrelin, an orexigenic hormone, is often associated with nutrient uptake; circulating levels of this hormone rise in preprandial periods and periods of fasting and then decrease during postprandial periods [82]. Studies of zebrafish found that fasting caused a significant increase in ghrelin expression in the gut and a significant reduction after re-feeding [81, 83]. A similar result was seen in goldfish (*Carassius auratus*), with variations in ghrelin expression being dependent on feeding state [84]. As the SH group showed numerically higher expression of ghrelin in the gut, and higher levels are typically associated with a fasting state in zebrafish [81], it is possible that the lower expression of ghrelin in the CI fish represented satiation due to increased feeding rate throughout the study [40]. It is important to note, however, that the zebrafish in this study were sampled approximately 18 to 20 h after receiving their last meal, and significant changes in ghrelin expression were not seen until 3–5 days of fasting in previous studies [81, 84].

Conversely, leptin is an anorexigenic hormone with a negative role in appetite, controlling diet and nutrient intake [85]. In goldfish, administering leptin via injection significantly reduced feed intake [86]. Leptin was found to be significantly reduced in zebrafish intestines after 12 and 24 h of fasting when compared to those that were sampled 2 h after feeding [87]. In another study, however, leptin increased through 48 h after feeding and only significantly decreased at the 192 h mark [83], suggesting that the timeframe for appetite-inhibitory feedback of this hormone can vary. The relatively lower numerical expression of leptin in the CI fish at only 18–20 h after feeding might suggest a faster return from the postprandial anorectic effects of leptin and may be another reason behind the increased feed intake that was observed.

Cholecystokinin (*CCK*) is another anorexigenic hormone that operates to signal satiety and trigger digestive enzyme secretion [88]. Additionally, *CCK* and leptin have been shown to act interdependently and leptin can cause an increase in *CCK* [86–87]. The numerically higher expression of both leptin and *CCK* genes in the gut of the SH fish may be a result of this interaction.

The hypothalamus represents the central command system controlling appetite and energy balance, and the monoaminergic system is largely involved not only in stress response but also in feed intake [89, 90]. Therefore, many complex interactions are likely involved in the expression of these appetite-related genes making it hard to directly pinpoint their influence on feed intake in this study. Additionally, analysis of the whole brain rather than specific regions (e.g. specific hypothalamic nuclei) may have influenced the results obtained.

Numerous studies have shown that SBM-based diets are associated with intestinal inflammation and reduced ingestion of feed; the degree of detrimental effects varies with species and amount of SBM included in the feed. Inclusion of SBM is typically limited in diets for carnivorous species (25–30%) as negative impacts on gut health are more prevalent at lower levels [91]. Detrimental effects on intestinal health were observed in Atlantic salmon (*Salmo salar*) at 10% dietary SBM inclusion and severe inflammation at 20% inclusion after only 3 days of feeding [35]. Similarly, 24% SBM dietary inclusion caused adverse effects on Japanese flounder (*Paralichthys olivaceus*) growth and health [92]. A study of turbot (*Scophthalmus maximus*) found that those fish fed SBM diets at rates of 26–54% inclusion showed dose-dependent increases in severity of intestinal inflammation, with progressively decreasing intestinal fold height and increased fusion of folds [91]. Individuals with increased intestinal inflammation also showed decreased growth performance and nutrient absorption [91]. Herbivores and omnivores are generally able to utilize higher dietary levels of SBM due to their overall better tolerance of plant feedstuffs [93]. There were no significant differences in weight gain or feed efficiency in juvenile redlip mullet (*Liza haematocheila*) fed 0, 25, 50, and 75% inclusion of SBM, however, the 75% SBM group did have relatively lower values than the other groups suggesting over 50% inclusion of SBM may have adverse effects [93]. Additionally, the 100% SBM group had the highest numerical daily feed intake but significantly lower feeding efficiency compared to the 0, 25, and 50% groups [93], exemplifying the higher feeding rate and lower utilization often observed in SBM-based diets. Similarly, studies of zebrafish found 50% inclusion of dietary SBM decreased the intestinal villi length, representative of an inflammatory reaction [36, 94]. Adept at utilizing both animal and plant feed sources and tolerant of higher levels of dietary SBM inclusion [36], zebrafish are a useful organism for modeling nutrition [95] and inflammatory mechanisms in both carnivorous and omnivorous species [96].

Interleukin-1beta (*il-1b*), interleukin-6 (*il-6*), and tumor necrosis factor alpha (*tnfa*) are pro-inflammatory cytokines that are important for modulating inflammatory reactions through innate and adaptive immune responses [97, 98]. The expression of these cytokines was examined because high-inclusion SBM diets often result in intestinal inflammation in a variety of fish species [32, 36, 91, 97, 98]. Previous studies found an increase in *il-1b* expression in zebrafish fed 30–50% inclusion SBM diets [36, 94, 97–99]. Expression of *il-1b* in our CI fish was significantly higher than in SH fish, possibly indicating an increase in inflammatory response within the intestine [100]. While both treatment groups in our study were fed with a diet based on 55.7% SBM, the CI group consumed significantly more on average than those in the SH group when measured as average milligrams consumed per fish. The increase in SBM diet consumption may have resulted in the increased expression of this pro-inflammatory cytokine. This increase can be seen in a study of seabass where the group that was fed a 50% SBM inclusion diet had the highest feeding rate: expression of *il-1b* in these fish was also significantly higher than in the FM and 75% SBM groups [56]. The increased expression of this pro-inflammatory cytokine may also explain the reduced feeding efficiency observed in the CI fish.

Interestingly, the expression of *il-6* and *tnfa* was not significantly different between treatment groups. Whereas past studies have shown that SBM diets tend to upregulate these pro-inflammatory cytokines [94, 97, 98], this effect was not observed in our study.

Although expression of *il-1b* was significantly higher in the CI fish, there were no morphological signs of intestinal inflammation at the end of the experiment. Histological analysis revealed that the villi length-to-width ratio in CI fish was significantly higher than in the SH fish. Villi length-to-width ratio is a common way of measuring potential inflammation and a lower ratio reduces surface area for absorption [37, 38]. A higher ratio usually indicates increased surface area in the intestine for nutrient absorption, which can translate to improved growth performance [37, 40]. Higher feed intake and increased expression of *il-1b* in the CI fish may have promoted morphological adaptation to SBM in the gut over time as *il-1b* signaling and immune-mediated inflammation are essential for tissue regeneration [101]. Changes in villi surface area are not just a consequence of a typical inflammatory diet but may also indicate an additive effect of social environment. In rodent studies, social stressors and chronic subordination were shown to increase inflammation of the gastrointestinal tract [102–104]. While not extensively studied in fish species, conflict for social dominance was found to cause cellular degeneration and atrophy of mucous epithelium in the stomachs of European eels (*Anguilla anguilla* L.) [105]. If social stress contributes to inflammation or damage of the gastrointestinal tract, perhaps CI fish were better able to mediate dietary-related inflammation without social conflicts as an additional stressor.

## Conclusion

The results from this study show that chronic isolation leads to increased feed intake of both marine and plant protein-based diets; it is important to note, however, that high FCR values suggest that increased feed intake did not translate to improved feed utilization. Although the mechanisms behind increased feed intake in this study are unclear, further investigation into effects of social interaction on feeding behavior may lead to useful tactics for promoting feed intake of less-palatable diets. Additionally, chronic isolation does not appear to negatively affect growth performance or intestinal morphology, nor does it alter expression of genes associated with monoaminergic function or appetite signaling after a period of 30 days. As reduced feed intake and growth performance are often used as indicators of stress, the increased feed intake and similar growth performance of CI fish compared to SH fish may support previous studies that suggest social isolation is not be inherently stressful to zebrafish.

## Supporting information

S1 File Zebrafish health analysis. This file contains the raw data for growth, feed intake, histological and gene expression analysis of the zebrafish used in this study.
(XLSX)
